# A Simple Taste Test for Clinical Assessment of Taste and Oral Somatosensory Function—The “Seven-iTT”

**DOI:** 10.3390/life13010059

**Published:** 2022-12-25

**Authors:** Mariano Mastinu, Michał Pieniak, Anne Wolf, Tomer Green, Antje Hähner, Masha Y. Niv, Thomas Hummel

**Affiliations:** 1 Smell and Taste Clinic, Department of Otorhinolaryngology, Technical University of Dresden, 01307 Dresden, Germany; 2 Institute of Psychology, University of Wroclaw, 50-529 Wrocław, Poland; 3 Institute of Biochemistry, Food Science and Nutrition, The Hebrew University of Jerusalem, Rehovot 7610001, Israel

**Keywords:** taste strips, gustatory assessment, sensory tests, oral somatosensory, astringency

## Abstract

Taste dysfunctions may occur, for example, after viral infection, surgery, medications, or with age. In clinical practice, it is important to assess patients’ taste function with rapidity and reliability. This study aimed to develop a test that assesses human gustatory sensitivity together with somatosensory functions of astringency and spiciness. A total of 154 healthy subjects and 51 patients with chemosensory dysfunction rated their gustatory sensitivity. They underwent a whole-mouth identification test of 12 filter-paper strips impregnated with low and high concentrations of sweet, sour, salty, bitter (sucrose, citric acid, NaCl, quinine), astringency (tannin), and spiciness (capsaicin). The percentage of correct identifications for high-concentrated sweet and sour, and for low-concentrated salty, bitter and spicy was lower in patients as compared with healthy participants. Interestingly, a lower identification in patients for both astringent concentrations was found. Based on the results, we proposed the Seven-iTT to assess chemo/somatosensory function, with a cut-off of 6 out of 7. The test score discriminated patients from healthy controls and showed gender differences among healthy controls. This quantitative test seems to be suitable for routine clinical assessment of gustatory and trigeminal function. It also provides new evidence on the mutual interaction between the two sensory systems.

## 1. Introduction

Taste perception can be affected by numerous disease and therapies. Viral infections, head traumas and surgical operation are the most frequent causes of taste dysfunction [1,2,3]. Additionally, medical causes such as neurodegenerative disease [4,5], diabetes [6], radio/chemotherapy, and metabolic disorders, or side effects of drugs [7] are also causes of taste decline [8]. The prevalence of taste loss in the general population can vary from 5% to 20%, which increases with age up to 33% [4,9].

Impairment in the taste can affect quality of life, food intake, and be a risk factor inducing depression [10,11]. Patients self-reporting taste deficit might overlap it with ortho/retronasal olfactory or trigeminal dysfunction. Thus, in clinical practice, assessment of human taste function is crucial to identify impairments in the chemosensation, to define the quality and quantity of gustatory loss, and separate it from possible olfactory dysfunctions. Normogeusia defines a physiological taste sensitivity, while hypogeusia and ageusia describe an impaired and a seriously damaged sense of taste, respectively. Available psychophysical tests are based on liquid dilutions dropped or sprayed in the tongue [12], tablet and edible wafer [13,14], which analyze the whole-mouth taste sensitivity. The most common identification test that assesses gustatory function is the Taste Strip Test. It is based on 16 filter paper strips, each one soaked with an increasing concentration of the four basic taste (sweet, sour, salty, and bitter) [15]. The test was validated for lateralization [16], in cross-cultural studies [17], and for evaluation of taste function in several diseases [18,19,20,21,22]. It can also be extended for the assessment of gustatory sensitivity for umami taste, the fifth basic taste often used to describe a meaty, savory flavor, by using 4 additional taste strips soaked with increasing concentration of monosodium glutamate [23].

However, the Taste Strip Test is not comprehensive for tactile, cooling, burning or astringent perceptions, which are known under the umbrella of somatosensory sensation. Touch, temperature and pain information are collected in the tongue by mechanoreceptors, thermoreceptors and nociceptors found in fungiform and filiform papillae [24,25,26], and carried to the brain by the trigeminal nerve. For example, astringency is the somatosensation marked by drying, roughing, and puckering of the oral surfaces that is experienced with consumption of polyphenol-rich foods (green tea, coffee, cocoa, berries, red wine) [27]. Although alum is used as a prototypical stimulus to evoke the astringent sensation, tannins are the most abundant astringent stimulus in food [28] that activate trigeminal stimulation [26]. In addition, capsaicin, which is the main trigeminal-activating compound in chili pepper [29], is applied to elicit the burning sensation of spiciness. Recently, oral tactile acuity was shown to be positive correlated to the density of fungiform papillae [30,31], and to be lower in patients with taste dysfunction compared to a control group [32], suggesting that impairments in taste have an impact on trigeminal sensitivity.

Up to date, spiciness is analyzed using capsaicin-impregnated filter paper strips or edible strips for threshold and suprathreshold analysis [33,34], whose threshold was different between patients after middle ear surgery and healthy controls [35]. Astringency sensation is mostly evaluated with liquid solutions [36,37], but without a validated test for trigeminal sensations, assessing impairments in the somatosensory function has been a challenge.

The aim of the present study was to develop a short taste test that evaluates the perception of four basic taste stimuli and includes astringent and spiciness sensation for an easy and quick assessment of taste and trigeminal function for clinical purpose.

## 2. Materials and Methods

### 2.1. Ethic Statement

The study was performed in accordance with the Declaration of Helsinki on Biomedical Studies Involving Human Subjects. Informed written consent was obtained from all participants prior to their inclusion in the study. The research protocol has been approved the Ethics Review Board at the University Clinic of the Technische Universität Dresden, application number BO-EK-25012021.

### 2.2. Participants

Two-hundred five individuals aged 18–81 years (M = 35.9, SD = 13.5 years; 131 females) were invited to participate in the study. Of these, 51 were patients of tertiary Smell and Taste Clinic who self-reported a chemosensory dysfunction. All participants received a complete otorhinolaryngological examination and a structured history was taken. The remaining study sample comprised 154 healthy controls as a reference group, recruited in Dresden (Germany) (*n* = 106; 67 females) and Jerusalem (Israel) (*n* = 48; 28 females). The groups were balanced in terms of gender distribution (*χ*^2^(1) = 1.32, *p* = 0.25, Odds Ratio = 0.67 [95% Confidence Intervals: 0.34; 1.33]) but patients were older than healthy controls (patients: *M* = 40.5, SD = 12.6 years; healthy controls: *M* = 34.4, SD = 13.5 years; *t* = 2.8, df = 203, *p* = 0.006 Cohen’s *d* = 0.45 [95% Confidence Intervals: 0.13; 0.77]). Subjects were excluded if any of this condition was present: pregnancy, allergy to substances used in the present study, unmedicated hypo/hyperthyreosis, uncontrolled diabetes mellitus, kidney failure, and significant cardiovascular issues.

### 2.3. Sensory Testing

To assess participants’ taste function, we used twelve filter paper strips (8 cm of length; 2 cm^2^ of tip area; 300 g/cm^2^) (Color Druck GmbH, Holzminden, Germany) impregnated with six tastants– sweet, sour, salty, bitter, astringent, and spicy (sucrose, CAS: 57-50-1 Sigma–Aldrich, Buchs, Switzerland: citric acid, CAS: 77-92-9 Sigma–Aldrich; sodium chloride, CAS: 7647-14-5 Sigma–Aldrich; quinine hydrochloride, CAS: 6119-47-7; tannin, Presque Isle Wine Cellars, North East, PA, USA; capsaicin 90% V/V ethanol, Kllinic-Apotheke, Dresden, Germany). For each of the taste quality, two intensities (high and low) were employed (Table 1). Taste strips were prepared regularly by soaking them in taste solutions for 30 s and drying them at room temperature according to previous works [16,38]. During each trial participants, who were refraining from eating or drinking anything except water for 1 h, were asked to open their mouth and extend their tongue. Strips were placed on the middle part of the tongue, approximately 1 cm from the tip of the tongue. Participants were then asked to close their mouth, taste the strip for few seconds, and identify the gustatory quality out of the six available options with a multiple forced-choice paradigm. Their responses were coded as 1 for correct responses (e.g., response ‘salty’ to a salty stimulus) or 0 for incorrect responses (e.g., response ‘salty’ to a sweet stimulus). Sum of correct identifications of these stimuli was used as a final score in the test. After presentation of each strip, participants rinsed their mouth with water until the sensation of the previews stimulus was gone. Strips were presented in a semi-randomized order, with trigeminal stimuli at last because of their long-lasting effect. The whole testing procedure lasted approximately 10 min. The subjective assessment of participant’s satisfaction with their sense of taste function was measured with one question: ‘How much are you satisfied with how your sense of taste functions from 0 to 100′. Additionally, participants self-rated their smell and taste sensitivity as well as sensitivity towards astringency and spiciness using 7-point Likert-type scale.

### 2.4. Statistical Analysis

All the statistical analyses were conducted with *jamovi* software (ver. 2.2.5) with the significance level set to *α* = 0.05. We employed non-parametric test due to uneven number of participants in two groups and non-normal distribution of the data. To verify the differences in satisfaction with sense of taste functioning and score in the taste test between patients and healthy controls we used a non-parametric Mann–Whitney U test. The same test was used to compare self-ratings of smell, taste, astringency and spiciness sensitivity. To verify if patients and healthy controls differed in taste stimuli recognition, we used a series of *χ*^2^ tests of association. The stimuli for which the recognition rate was different between patients and healthy subjects were employed to the final version of the test. We took different scores in the taste test as cut-off criteria to divide participants into groups of healthy individuals and patients with a chemosensory dysfunction and calculated test sensitivity, specificity and Cohen’s kappa (*κ*) indicator of interrater reliability [39]. Subjective satisfaction with taste function and self-rated taste sensitivity were compared with score in the taste test using Spearman’s correlation analysis. Finally, Mann–Whitney U test was also used to verify differences in taste score according to gender in healthy controls and patients. For all Mann–Whitney U tests we provided rank biserial correlation (r) as an effect size estimation. Rank biserial correlation values range between 0 and 1 where greater correlation indicates greater difference between ranks of the compared groups [40]. Effect size for χ^2^ tests of association was expressed with Odds Ratio (OR) followed by 95% Confidence Intervals (CI) in square brackets. OR equal 1 suggest similar chance of correct recognition of a taste strip between patients and healthy controls. OR lower than 1 indicates increased prevalence of incorrect recognition of a taste strip in the patients’ cohort.

## 3. Results

**Figure 1:**
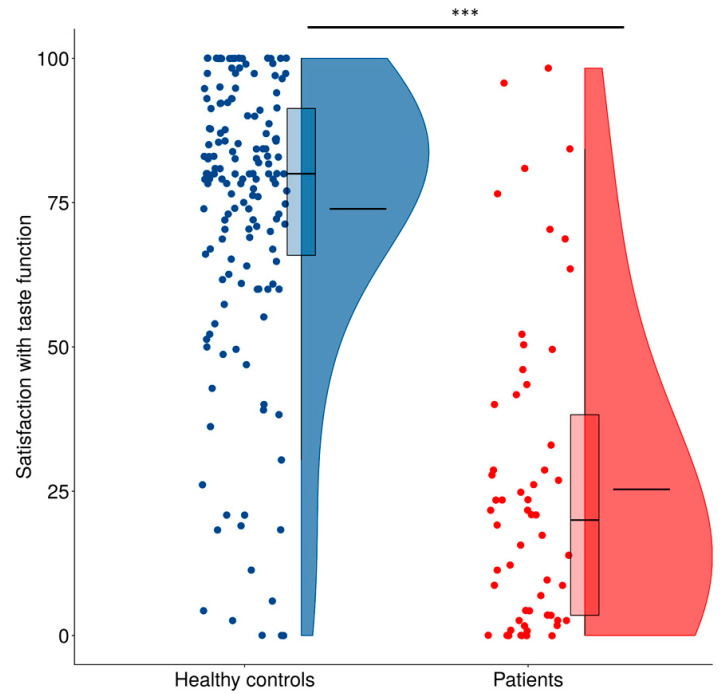
Ratings of satisfaction with taste function of healthy controls and patients. Flat lines within the boxplots denote median and boxes represent the range between 25th and 75th percentile. Flat lines within the violin plots denote mean. Statistically significant difference is marked with asterisks. *** means *p* = 0.001.

We found statistically significant differences between patients with subjective chemosensory dysfunction and healthy controls in their satisfaction with sense of taste function. The analysis revealed that the satisfaction level was significantly lower in patients (*Me = 15.7*, *M* = 22.5, SD = 24.9) as compared with their healthy counterparts (*Me* = 80.0, *M* = 73.9, SD = 24.6) (*U* =804, *p* < 0.001, *r* = 0.80; Figure 1).

**Figure 2:**
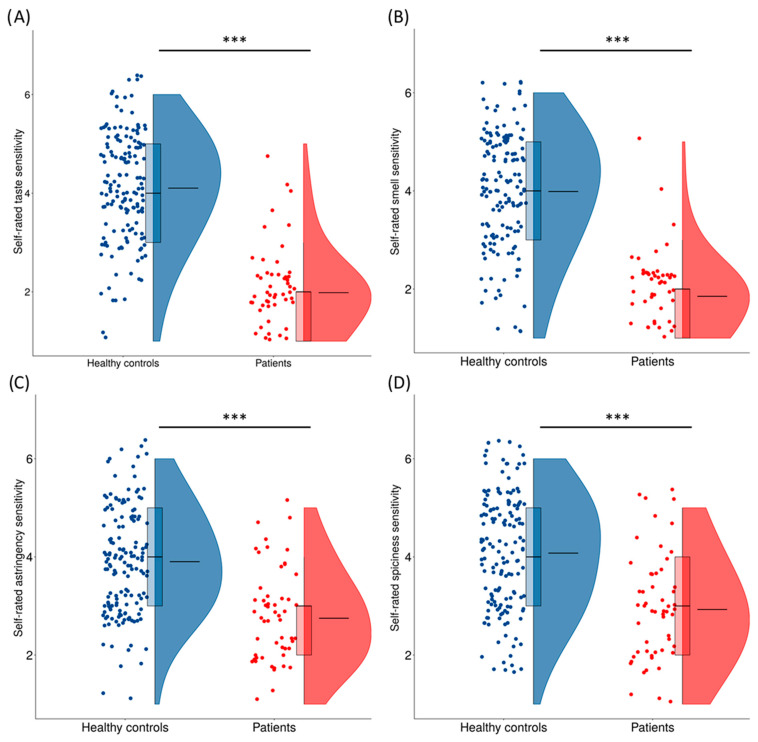
Self-ratings of taste (**A**), smell (**B**), astringency (**C**), and spiciness (**D**) sensitivity among healthy controls and patients. Flat line within the boxplot denotes median and boxes represent the range between 25th and 75th percentile. Flat line within the violin plot denotes mean. Statistically significant differences are marked with asterisks. *** means *p* < 0.001.

Further analyses showed that patients systematically rated their taste (*U* = 679, *p* < 0.001, *r* = 0.83) and smell (*U* = 748, *p* < 0.001, *r* = 0.81) sensitivity as lower than healthy controls did. The same effect has been observed also for the sensitivity towards astringent (*U* = 1718, *p* < 0.001, *r* = 0.56) and spicy (*U* = 1826, *p* < 0.001, *r* = 0.54) stimuli. The distributions of self-ratings are presented in Figure 2.

**Figure 3:**
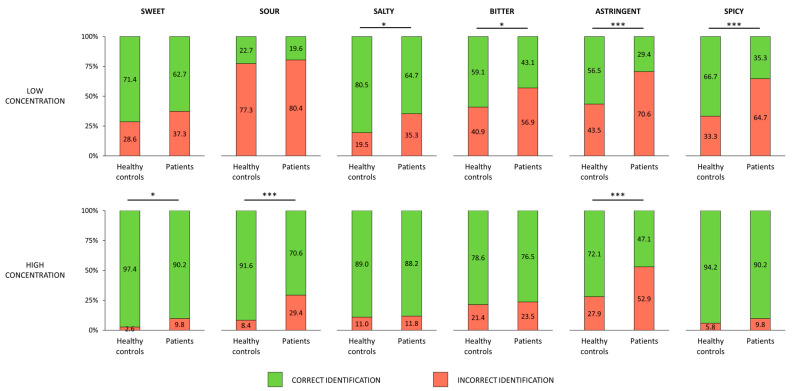
Proportions of correct and incorrect identifications of different types of taste stimuli among healthy controls and patients. Statistically significant differences in proportion in correct identification between the patients and healthy controls are marked with asterisks. * means *p* < 0.05 while *** *p* ≤ 0.001.

A series of *χ*^2^ tests of association demonstrated that proportion of individuals that correctly recognized the tested taste quality was lower in patients than in healthy controls. This finding was consistent across all employed taste qualities; however, it was observed for different concentrations depending on the taste quality. For instance, for high-concentrated sweet (*χ*^2^ (1) = 4.74; *p* = 0.029, OR = 0.25 [CI: 0.06; 0.95]) and sour (*χ*^2^ (1) = 14.3; *p* < 0.001, OR = 0.22 [CI: 0.1; 0.51]) tastes the proportion of correct identification was lower in patients group as compared with healthy participants, but remained similar for these tastes in low concentration (sweet: *χ*^2^ (1) = 1.36; *p* = 0.244, OR = 0.67 [CI: 0.35; 1.31]; sour: *χ*^2^ (1) = 0.218; *p* = 0.641, OR = 0.83 [CI: 0.38; 1.82]). For salty and bitter—in low concentrations patients recognized these tastes less frequently than healthy controls (salty: *χ*^2^ (1) = 5.34; *p* = 0.021, OR = 0.44 [CI: 0.22; 0.89]; bitter: *χ*^2^ (1) = 3.94; *p* = 0.047, OR = 0.53 [CI: 0.28; 1]), but in high concentrations the proportion of correct identification was similar across groups (salty: *χ*^2^ (1) = 0.02; *p* = 0.89, OR = 0.93 [CI: 0.35; 2.5]; bitter: *χ*^2^(1) = 0.097; *p* = 0.75, OR = 0.89 [CI: 0.42; 1.89]). Astringency was the only quality that has been identified less frequently by patients as compared with controls in both high (*χ*^2^ (1) = 10.7; *p* = 0.001, OR = 0.34 [CI: 0.18; 0.66]) and low (*χ*^2^ (1) = 0.11,2; *p* < 0.001, OR = 0.32 [CI: 0.16; 0.63]) concentrations. The spicy taste quality was recognized equally well by patients and healthy counterparts in high concentrations (*χ*^2^ (1) = 0.94; *p* = 0.33, OR = 0.57 [CI: 0.18; 1.79]), but in low concentration patients recognized it less often than healthy controls (*χ*^2^ (1) = 15.5; *p* < 0.001, OR = 0.27 [CI: 0.14; 0.53]). The proportions of correct and incorrect recognitions of each taste quality in high and low concentrations are presented in Figure 3.

**Figure 4:**
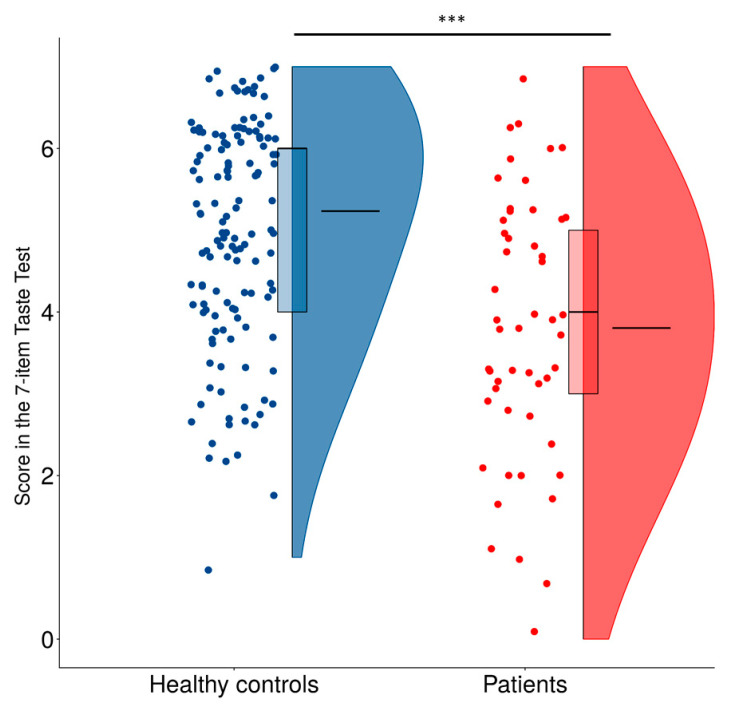
Scores in taste test of healthy controls and patients. Flat lines within the boxplots denote median and boxes represent the range between 25th and 75th percentile. Flat lines within the violin plots denote mean. Statistically significant difference is marked with asterisks. *** means *p* < 0.001.

Overall, patients (*Me* = 4, *M* = 3.8, SD = 1.6) scored significantly lower in the taste test as compared with healthy controls (*Me* = 6, *M* = 5.24, SD = 1.5; *U* = 2007, *p* < 0.001, r = 0.49; Figure 4). We found that using score of 6 correct identifications in the 7-item taste test as a cut-off criterion for chemosensory dysfunction leads to 84.3% test sensitivity and 51.0% test specificity (Cohen’s *κ* = 0.25, fair agreement). These coefficients were worse for score of 5 correct identifications (sensitivity: 60.8%, specificity: 69.9%, Cohen’s *κ* = 0.26) and 7 correct identifications (sensitivity: 98.0%, specificity: 22.2%, Cohen’s *κ* = 0.11) as cut-off criteria. Score in the taste test was positively correlated with self-reported sensitivity of taste (*r* = 0.28, *p* < 0.001), which was significant only in healthy controls (*r* = 0.19, *p* = 0.022) but not in patients, and with satisfaction for taste function (*r* = 0.36, *p* < 0.001), which was significant in patients (*r* = 0.29, *p* = 0.039) but not in healthy controls.

In addition, Mann–Whitney test revealed that score in the test was significantly different according to gender in healthy controls (*U* = 679, *p* < 0.001, *r* = 0.18), with female scoring a higher identification than men score, whereas it was not different in patients (*p* > 0.05).

## 4. Discussion

The present study aimed to establish an extensive method for the evaluation of taste and oral trigeminal functions, with the characteristic to be brief and easy to apply, comprehensive, and with a long shelf life. Twelve strips impregnated with chemosensory stimuli (including sweet, salty, sour, bitter, astringent and spicy stimuli) were used to assess the general chemosensory function in a group of healthy controls and in patients who expressed an impairment in their taste perception.

Firstly, we showed that patients had significantly lower satisfaction with their taste function, and their self-rated taste, smell, astringency and spiciness sensitivities were significantly lower than the ones rated by healthy controls. These self-reported ratings distinguished well between the two groups. These data are in accordance with previous studies that showed a positive relationship between subjects’ self-assessment and taste identification performance [41,42], whereas some other studies have not found this correlation on patients [43,44,45,46] or healthy subjects [47]. This divergence may be due to a common mistake in discrimination between decreased taste and retronasal olfaction [1], thus some patients who complain taste loss might not respond accurately to this type of questionnaire.

We analyzed the differences in strip identification between the two groups. Patients who expressed impairments in taste had lower identification scores than healthy controls for low concentration of salty, bitter and spicy sensations, which were sufficient to significantly differentiate the two groups, while high concentration of sweet and salty were required for this goal. In the past, epidemiological studies based on a large number of healthy subjects showed that sweet was the most correctly identified taste sensation, followed by salty, bitter and sour [8,48]. These data were confirmed in our cohort. Additionally, the low concentration that we used for sweet taste was highly identified by both healthy controls and patients, endorsing that sweet is easily detectable in patients with taste impairments.

On the other hand, the two astringent concentrations were able to discriminate healthy controls from patients. In this study, patients self-reported a taste disturbance, and a lower astringent sensitivity, that might reflect a generalized impairment also in the somatosensory function. Bogdanov and colleagues demonstrated that patients with taste impairments had lower acuity also in their somatosensory sensitivity in a 3D-letter identification test [32], which was correlated with fungiform papillae density [30], whereas in another study, texture acuity was higher in subjects with high sensitivity for the bitter compound 6-n-propylthiouracil (PROP) [49]. Additionally, the intensity of astringency was rated as lower in PROP non-sensitive subjects according to gender [50,51] which are known to have lower density of fungiform papillae in the anterior two-thirds of the dorsal tongue surface and lower taste sensitivity compared to the sensitive phenotype [52,53]. In a recent study on a group of COVID-19 patients, 85% of whom reporting a taste impairment, showed expressed alteration also in mouthfeel and temperature sensation in 58% and 25% of them, respectively [54]. Furthermore, in this study patients had a lower identification score for low concentration of capsaicin-evoked sensation. Our results, together with the present literature, allow us to speculate that gustatory and tactile sensitivities strongly interact at a functional level. As so, an impairment in chemosensation might negatively affect the somatosensory sensation.

Based on these results, we proposed a 7-item chemosensory test (Seven-iTT), which includes strips that had different identification frequencies between healthy controls and patients: high concentration of sweet and sour, lower concentration of salty, bitter and spicy, and both concentrations of the astringent stimulus. We used Cohen’s kappa indicator to calculate sensitivity and specificity of the test to divide the two groups for their chemosensory dysfunction based on different cut-off criteria. Using 5 correct identification out of 7, a low sensitivity and specificity were found whereas setting the cut-off at 7 increased the sensitivity at the cost of its specificity, as expected. The proposed criterion to define a possible chemosensory impairment was set at 6 out of 7, which was sufficient to significantly distinguish patients from the healthy controls as shown in Figure 4. Additional studies are needed to investigate the validity of the test in patients with specific taste disorders.

Our study additionally confirmed previous findings on a higher identification in women compared to men for suprathreshold taste stimuli [13,16,55]. It has been hypothesized that this difference is driven by a hormonal effect that finally affects chemosensory function [56]. This difference was significant only in the healthy controls but not in patients, implying that taste impairment tends to reduce and confuse the sensory difference between genders. 

The present data refers only to the whole mouth sensation of taste and trigeminal sensation. Indeed, we asked subjects to close their month while tasting the strip. However, the small dimension of strips allows the Seven-iTT to be separately applied in the left or in the right side of the anterior third of the tongue for lateral evaluation. This will be a clear advantage in clinical settings to assess taste and somatosensory deficits resulting, for example, from lateralized lesions of the chorda tympani [57]. Further studies will investigate the lateralized use of this test to assess regional function and assess unilaterally chemosensory dysfunction in the left or right side of the tongue.

Taste sensitivities for umami and fatty acid were not analyzed in this study, therefore they were not included in the final version of the test. Despite the sensitivity to umami varies among individual, and it was shown to exhibit taste laterality in cerebral processing [58], this taste concept has been found to be difficult to explain to the European population that we tested [59]. On the other side, fatty-acid sensitivity can be examined by mean of test that implies oleic acid as a prototype stimulus [60]. The problem related to the stimulus’ thermal instability require preparing it real time, affecting the duration of the procedure and the shelf life of the strips.

Results from the present study on the direct relationships between Seven-iTT score with self-reported sensitivity of taste and with satisfaction for taste function, together with gender differences, confirm that the proposed test is a reliable method for the assessment of chemosensory functions.

## 5. Conclusions

The present results provide a fast, reliable and extensive version of Taste Strip test for the evaluation of basic tastes, and trigeminal function. It appears to require little time, to be easy to use and suitable to routine clinical investigations.

## Figures and Tables

**Table 1:** Taste stimuli used in the study and their concentration.

Taste Quality	Substance	Low Concentration (g/mL)	High Concentration (g/mL)
Sweet	Sucrose	0.05	0.4
Sour	Citric acid	0.05	0.3
Salty	Sodium chloride	0.016	0.25
Bitter	Quinine hydrochloride	0.0004	0.006
Astringent	Tannin	0.1	0.2
Spicy	Capsaicin	2.47 × 10^−5^	2.47 × 10^−4^

## Data Availability

Anonymized data will be made available upon reasonable request to the corresponding author.

## References

[1] Deems D.A., Doty R.L., Settle R.G., Moore-Gillon V., Shaman P., Mester A.F., Kimmelman C.P., Brightman V.J., Snow J.B. (1991). Smell and taste disorders, a study of 750 patients from the University of Pennsylvania Smell and Taste Center. Arch. Otolaryngol. Head Neck Surg..

[2] Sakashita S., Takayama K., Nishioka K., Katoh T. (2004). Taste disorders in healthy “carriers” and “non-carriers” of *Candida albicans* and in patients with candidosis of the tongue. J. Dermatol..

[3] Fark T., Hummel C., Hahner A., Nin T., Hummel T. (2013). Characteristics of taste disorders. Eur. Arch. Otorhinolaryngol..

[4] Doty R.L., Hawkes C.H. (2019). Chemosensory dysfunction in neurodegenerative diseases. Handb. Clin. Neurol..

[5] Melis M., Haehner A., Mastinu M., Hummel T., Barbarossa I.T. (2021). Molecular and Genetic Factors Involved in Olfactory and Gustatory Deficits and Associations with Microbiota in Parkinson’s Disease. Int. J. Mol. Sci..

[6] Naka A., Riedl M., Luger A., Hummel T., Mueller C.A. (2010). Clinical significance of smell and taste disorders in patients with diabetes mellitus. Eur. Arch. Otorhinolaryngol..

[7] Douglass R., Heckman G. (2010). Drug-related taste disturbance: A contributing factor in geriatric syndromes. Can. Fam. Physician.

[8] Welge-Lüssen A., Dörig P., Wolfensberger M., Krone F., Hummel T. (2011). A study about the frequency of taste disorders. J. Neurol..

[9] Sergi G., Bano G., Pizzato S., Veronese N., Manzato E. (2017). Taste loss in the elderly: Possible implications for dietary habits. Crit. Rev. Food Sci. Nutr..

[10] Merkonidis C., Grosse F., Ninh T., Hummel C., Haehner A., Hummel T. (2015). Characteristics of chemosensory disorders—Results from a survey. Eur. Arch. Oto-Rhino-Laryngol..

[11] Risso D., Drayna D., Morini G. (2020). Alteration, Reduction and Taste Loss: Main Causes and Potential Implications on Dietary Habits. Nutrients.

[12] Vennemann M.M., Hummel T., Berger K. (2008). The association between smoking and smell and taste impairment in the general population. J. Neurol..

[13] Ahne G., Erras A., Hummel T., Kobal G. (2000). Assessment of gustatory function by means of tasting tablets. Laryngoscope.

[14] Hummel T., Erras A., Kobal G. (1997). A test for the screening of taste function. J. Rhinol..

[15] Mueller C., Kallert S., Renner B., Stiassny K., Temmel A.F., Hummel T., Kobal G. (2003). Quantitative assessment of gustatory function in a clinical context using impregnated “taste strips”. J. Rhinol..

[16] Landis B.N., Welge-Luessen A., Bramerson A., Bende M., Mueller C.A., Nordin S., Hummel T. (2009). “Taste Strips”—A rapid, lateralized, gustatory bedside identification test based on impregnated filter papers. J. Neurol..

[17] Ribeiro J.C., Chaves M., Chaves C., Lemos L., Silva E.D., Paiva A., Hummel T. (2016). Cross-cultural validation of a taste test with paper strips. Eur. Arch. Oto-Rhino-Laryngol..

[18] Mueller C.A., Khatib S., Landis B.N., Temmel A.F., Hummel T. (2007). Gustatory function after tonsillectomy. Arch. Otolaryngol. Head Neck Surg..

[19] Melis M., Mastinu M., Sollai G., Paduano D., Chicco F., Magri S., Usai P., Crnjar R., Tepper B.J., Barbarossa I.T. (2020). Taste Changes in Patients with Inflammatory Bowel Disease: Associations with PROP Phenotypes and polymorphisms in the salivary protein, Gustin and CD36 Receptor Genes. Nutrients.

[20] Cecchini M.P., Osculati F., Ottaviani S., Boschi F., Fasano A., Tinazzi M. (2014). Taste performance in Parkinson’s disease. J. Neural. Transm..

[21] Heckmann J.G., Stossel C., Lang C.J., Neundorfer B., Tomandl B., Hummel T. (2005). Taste disorders in acute stroke: A prospective observational study on taste disorders in 102 stroke patients. Stroke.

[22] Melis M., Pintus S., Mastinu M., Fantola G., Moroni R., Pepino M.Y., Barbarossa I.T. (2021). Changes of Taste, Smell and Eating Behavior in Patients Undergoing Bariatric Surgery: Associations with PROP Phenotypes and Polymorphisms in the Odorant-Binding Protein OBPIIa and CD36 Receptor Genes. Nutrients.

[23] Mueller C.A., Pintscher K., Renner B. (2011). Clinical test of gustatory function including umami taste. Ann. Otol. Rhinol. Laryngol..

[24] Des Gachons C.P., Uchida K., Bryant B., Shima A., Sperry J.B., Dankulich-Nagrudny L., Tominaga M., Smith A.B., Beauchamp G.K., Breslin P.A. (2011). Unusual pungency from extra-virgin olive oil is attributable to restricted spatial expression of the receptor of oleocanthal. J. Neurosci..

[25] Moayedi Y., Michlig S., Park M., Koch A., Lumpkin E.A. (2021). Somatosensory innervation of healthy human oral tissues. J. Comp. Neurol..

[26] Schöbel N., Radtke D., Kyereme J., Wollmann N., Cichy A., Obst K., Kallweit K., Kletke O., Minovi A., Dazert S. (2014). Astringency is a trigeminal sensation that involves the activation of G protein–coupled signaling by phenolic compounds. Chem. Senses.

[27] Lee C.B., Lawless H.T. (1991). Time-course of astringent sensations. Chem. Senses.

[28] Chung K.T., Wong T.Y., Wei C.I., Huang Y.W., Lin Y. (1998). Tannins and human health: A review. Crit. Rev. Food Sci. Nutr..

[29] Smutzer G., Devassy R.K. (2016). Integrating TRPV1 Receptor Function with Capsaicin Psychophysics. Adv. Pharmacol. Sci..

[30] Bangcuyo R.G., Simons C.T. (2017). Lingual tactile sensitivity: Effect of age group, sex, and fungiform papillae density. Exp. Brain Res..

[31] Essick G.K., Chopra A., Guest S., McGlone F. (2003). Lingual tactile acuity, taste perception, and the density and diameter of fungiform papillae in female subjects. Physiol. Behav..

[32] Bogdanov V., Reinhard J., McGlone F., Haehner A., Simons C.T., Hummel T. (2021). Oral Somatosensory Sensitivity in Patients with Taste Disturbance. Laryngoscope.

[33] Just T., Pau H.W., Steiner S., Hummel T. (2007). Assessment of oral trigeminal sensitivity in humans. Eur. Arch. Oto-Rhino-Laryngol..

[34] Smutzer G., Jacob J.C., Tran J.T., Shah D.I., Gambhir S., Devassy R.K., Tran E.B., Hoang B.T., McCune J.F. (2018). Detection and modulation of capsaicin perception in the human oral cavity. Physiol. Behav..

[35] Just T., Steiner S., Strenger T., Pau H.W. (2007). Changes of oral trigeminal sensitivity in patients after middle ear surgery. Laryngoscope.

[36] Roukka S., Puputti S., Aisala H., Hoppu U., Seppa L., Sandell M.A. (2021). The Individual Differences in the Perception of Oral Chemesthesis Are Linked to Taste Sensitivity. Foods.

[37] Wang M., Septier C., Brignot H., Martin C., Canon F., Feron G. (2022). Astringency Sensitivity to Tannic Acid: Effect of Ageing and Saliva. Molecules.

[38] Zhu Y., Hummel T. (2021). Assessment of Taste Function.

[39] McHugh M.L. (2012). Interrater reliability: The kappa statistic. Biochem. Med..

[40] Kerby D.S. (2014). The simple difference formula: An approach to teaching nonparametric correlation. Compr. Psychol..

[41] Soter A., Kim J., Jackman A., Tourbier I., Kaul A., Doty R.L. (2008). Accuracy of self-report in detecting taste dysfunction. Laryngoscope.

[42] Park Y.-J., Kho H.-S. (2022). Relationship between subjective taste sensations and taste strip test in patients with taste disorders with and without burning mouth syndrome. J. Dent. Sci..

[43] Liu D.T., Besser G., Renner B., Seyferth S., Hummel T., Mueller C.A. (2020). Retronasal olfactory function in patients with smell loss but subjectively normal flavor perception. Laryngoscope.

[44] Li Z., Stolper S., Draf J., Haehner A., Hummel T. (2022). Smell, taste and trigeminal function: Similarities and differences between results from home tests and examinations in the clinic. J. Rhinol..

[45] Steinbach S., Reindl W., Kessel C., Ott R., Zahnert T., Hundt W., Heinrich P., Saur D., Huber W. (2010). Olfactory and gustatory function in irritable bowel syndrome. Eur. Arch. Oto-Rhino-Laryngol..

[46] Grasl S., Janik S., Wiederstein S., Haymerle G., Renner B., Mueller C.A. (2022). Chemosensory Functions After Glossectomy—A Cross-Sectional Pilot Study. Laryngoscope.

[47] Green T., Wolf A., Oleszkiewicz A., Aronis A., Hummel T., Pepino M.Y., Niv M.Y. (2022). Subjective assessment and taste strips testing of gustatory function, at home, and in the lab. bioRxiv.

[48] Schumm L.P., McClintock M., Williams S., Leitsch S., Lundstrom J., Hummel T., Lindau S.T. (2009). Assessment of sensory function in the National Social Life, Health, and Aging Project. J. Gerontol. B Psychol. Sci. Soc. Sci..

[49] Essick G.K., Chen C.C., Kelly D.G. (1999). A letter-recognition task to assess lingual tactile acuity. J. Oral Maxillofac. Surg..

[50] Melis M., Yousaf N.Y., Mattes M.Z., Cabras T., Messana I., Crnjar R., Tomassini Barbarossa I., Tepper B.J. (2017). Sensory perception of and salivary protein response to astringency as a function of the 6-n-propylthioural (PROP) bitter-taste phenotype. Physiol. Behav..

[51] Yousaf N.Y., Tepper B.J. (2022). The Effects of Cranberry Polyphenol Extract (CPE) Supplementation on Astringency and Flavor Perception as a Function of PROP Taster Status and Other Individual Factors. Int. J. Environ. Res. Public Health.

[52] Melis M., Atzori E., Cabras S., Zonza A., Calo C., Muroni P., Nieddu M., Padiglia A., Sogos V., Tepper B.J. (2013). The gustin (CA6) gene polymorphism, rs2274333 (A/G), as a mechanistic link between PROP tasting and fungiform taste papilla density and maintenance. PLoS ONE.

[53] Sollai G., Melis M., Pani D., Cosseddu P., Usai I., Crnjar R., Bonfiglio A., Tomassini Barbarossa I. (2017). First objective evaluation of taste sensitivity to 6-n-propylthiouracil (PROP), a paradigm gustatory stimulus in humans. Sci. Rep..

[54] van Elst J.M., Boesveldt S., Vissink A., Jager-Wittenaar H., Reyners A.K., de Haan J.J. (2022). Subjective Mouthfeel and Temperature Alterations in COVID-19 Patients Six to Ten Months After Diagnosis. Chemosens. Percept..

[55] Gudziol H., Hummel T. (2007). Normative values for the assessment of gustatory function using liquid tastants. Acta Oto-Laryngol..

[56] Doty R.L., Cameron E.L. (2009). Sex differences and reproductive hormone influences on human odor perception. Physiol. Behav..

[57] Maeda E., Katsura H., Nin T., Sakaguchi-Fukunaga A., Mishiro Y., Sakagami M. (2018). Change of somatosensory function of the tongue caused by chorda tympani nerve disorder after stapes surgery. Laryngoscope.

[58] Iannilli E., Singh P.B., Schuster B., Gerber J., Hummel T. (2012). Taste laterality studied by means of umami and salt stimuli: An fMRI study. Neuroimage.

[59] Cecchini M.P., Knaapila A., Hoffmann E., Boschi F., Hummel T., Iannilli E. (2019). A cross-cultural survey of umami familiarity in European countries. Food Qual. Prefer..

[60] Melis M., Mastinu M., Arca M., Crnjar R., Tomassini Barbarossa I. (2018). Effect of chemical interaction between oleic acid and L-Arginine on oral perception, as a function of polymorphisms of CD36 and OBPIIa and genetic ability to taste 6-n-propylthiouracil. PLoS ONE.

